# Molecular Mechanisms Driving the *In Vivo* Development of KPC-71-Mediated Resistance to Ceftazidime-Avibactam during Treatment of Carbapenem-Resistant Klebsiella pneumoniae Infections

**DOI:** 10.1128/mSphere.00859-21

**Published:** 2021-12-22

**Authors:** Xi Li, Huanhuan Ke, Wenhao Wu, Yuexing Tu, Hua Zhou, Yunsong Yu

**Affiliations:** a Centre of Laboratory Medicine, Zhejiang Provincial People’ s Hospital, People’ s Hospital of Hangzhou Medical College, Hangzhou, Zhejiang, China; b Department of Biophysics and Department of Pathology, Sir Run Run Shaw Hospital, Zhejiang University School of Medicine, Hangzhou, China; c Department of Rehabilitation Medicine, Zhejiang Provincial People’s Hospital, People’s Hospital of Hangzhou Medical College, Hangzhou, Zhejiang, China; d Department of Respiratory and Critical Care Medicine, The First Affiliated Hospital, School of Medicine, Zhejiang University, Hangzhou, Zhejiang, China; e Key Laboratory of Microbial Technology and Bioinformatics of Zhejiang Province, Hangzhou, Zhejiang, China; f Department of Infectious Diseases, Sir Run Run Shaw Hospital, College of Medicine, Zhejiang University, Hangzhou, Zhejiang, China; g Regional Medical Center for National Institute of Respiratory Diseases, Sir Run Run Shaw Hospital, Zhejiang University School of Medicine, Hangzhou, China; Antimicrobial Development Specialists, LLC

**Keywords:** CRKP, KPC-71, CZA resistance, fitness

## Abstract

Here, we characterized the mechanisms resulting in the development of KPC-71-mediated resistance to ceftazidime-avibactam (CZA) during treatment of carbapenem-resistant Klebsiella pneumoniae (CRKP) infections. CZA-susceptible and CZA-resistant K. pneumoniae strains, namely, KP357 and KP697, were isolated from the same patient. Whole-genome sequencing revealed that KP357 and KP697 belonged to the ST11 type and KP697 strain possessed a mutation in the plasmid-borne *bla*_KPC-2_ gene. Compared to KPC-2, this *bla*_KPC_ gene (*bla*_KPC-71_) showed a mutated nucleotide and an insertion of 3 nucleotides at positions 542 to 545, which resulted in a variant with the subsequent insertion of a serine between the Ambler positions 182 and 183. This plasmid, carrying *bla*_KPC-71_, successfully transformed its CZA-resistant phenotype to Escherichia coli DH5α. Cloning and expression of *bla*_KPC-71_ in E. coli DH5α demonstrated that KPC-71 resulted in a 16-fold increase in the MIC value for CZA. Kinetic parameters showed that KPC-71, compared to wild-type KPC-2, exhibited a lower (∼13-fold) *K_m_* with ceftazidime and a higher (∼14-fold) 50% inhibitory concentration with avibactam. In addition, both *bla*_KPC-2_ and *bla*_KPC-71_ gene expression have a negative impact on fitness. In conclusion, we detected a novel KPC variant, KPC-71, in a clinical ST11 CRKP strain resulting in CZA resistance development during treatment. The KPC-71 enzyme was associated with a higher affinity toward ceftazidime and a reduced sensitivity to avibactam, conferring resistance to CZA. Considering the wide application of CZA, clinicians should pay attention to the risk of the development of CZA resistance in CRKP strains under treatment pressure.

**IMPORTANCE** In this study, we report an ST11-type clinical CRKP isolate that produces KPC-71, a novel plasmid backbone KPC variant that confers the development of CZA resistance during treatment. Furthermore, we reveal that resistance to CZA is mediated by the 182S insertion mutation in the KPC enzyme, which increases ceftazidime affinity and decreases avibactam inhibition. In addition, KPC-71 has reduced hydrolysis activity, which leads to susceptibility to carbapenems. To the best of our knowledge, this is a novel KPC-2 variant conferring resistance to CZA and the first report of its emergence. Considering the widespread presence of the ST11 CRKP strain in China, clinicians should pay attention to the risk of the development of CZA resistance in CRKP strains under treatment pressure.

## INTRODUCTION

Carbapenem-resistant *Enterobacterales* have rapidly spread worldwide and have become one of the major threats to public health (1). Ceftazidime-avibactam (CZA) is an effective alternative antibiotic for clinical treatment against bacteria producing several classes of β-lactamases, such as AmpC, extended-spectrum β-lactamases (ESBL), KPC-2, and OXA-48-like variants (2). In China, carbapenem resistance in Klebsiella pneumoniae is caused mainly by the production of the KPC enzyme (3). CZA treatment of carbapenem-resistant K. pneumoniae (CRKP) bacteremia is associated with significantly higher clinical survival rates than other regimens (4, 5). However, with gradually increasing usage of CZA, acquired resistance has been reported in patients with or without a history of CZA therapy (6–12).

Currently, the CZA resistance mechanisms primarily involve enzymes leading to antibiotic inactivation, chemical modification of antibiotic targets or substitution target expression, and changes in cell permeability or efflux pump expression (13). Among these mechanisms, KPC enzyme variants are a common cause of CZA resistance besides production of metallo-beta-lactamases (MBLs). To date, 90 variants of the *bla*_KPC_ gene have been identified among Gram-negative bacteria worldwide, including more than 30 conferring CZA resistance (https://www.ncbi.nlm.nih.gov/pathogens/isolates#/refgene/KPC). Amino acid mutations of the *bla*_KPC_ gene have been identified in mainly one of four loops (loop Leu102 to Ser106, Ω-loop Arg164 to Asp179, or loops Cys238 to Thr243 and Ala267 to Ser275) (14). Emergence of CZA resistance causes limitations to clinical treatment of CRKP. However, CZA resistance mechanisms not associated with the loop region of KPC are largely unknown.

In the present study, two CRKP ST11 clinical strains that presented the phenotype transformation from CZA susceptibility to the development of CZA resistance during treatment were isolated. CZA resistance was mediated by a novel KPC variant, KPC-71, that possessed a 182S insertion in its protein sequence compared to that of KPC-2. The KPC-71 enzyme was associated with a higher affinity toward ceftazidime and a reduced sensitivity to avibactam, conferring resistance to CZA.

## RESULTS

### Characteristic of clinical K. pneumoniae isolates.

**FIG 1:**
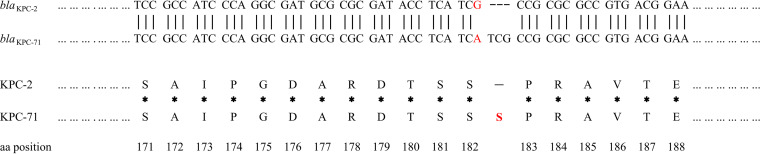
Amplicon alignments between *bla*_KPC-2_ and *bla*_KPC-71_ in nucleotide and amino acid (aa) sequences surrounding the mutation. One mutated nucleotide and an insertion of 3 nucleotides were identified at the *bla*_KPC-71_ gene compared to *bla*_KPC-2_, which led to serine between amino acid sequence positions 182 and 183 of the KPC-2 protein. The red letters represent inconsistent bases and amino acids. Dotted line, common sequence; broken line, insertion of three nucleotides; *, common amino acid; boldface font, insertion of an amino acid. A, Ala; D, Asp; E, Glu; G, Gly; I, Ile; P, Pro; R, Arg; S, Ser; T, Thr; V, Val; Y, Tyr.

The K. pneumoniae ancestral isolate (KP357) was resistant to amoxicillin-clavulanic acid, cefepime, ceftazidime, ertapenem, imipenem, meropenem, amikacin, ciprofloxacin, and tigecycline but susceptible to colistin and CZA (Table 1). The K. pneumoniae descendant isolate (KP697) showed an almost consistent susceptibility pattern, with the exception of CZA resistance and reversion to imipenem and meropenem susceptibility (Table 1). Whole-genome sequencing analysis revealed that the two strains both belonged to the ST11 type and carried multiple resistance genes, including *bla*_TEM-1_, *bla*_LAP-2_, *bla*_CTX-M-65_, *aadA2*, *rmtb*, *dfrA14*, *qnrS1*, *catA2*, *fosA*, *tet*(A), and *sul2.* Notably, whole-genome comparison analysis further revealed that the two K. pneumoniae strains contained one mutation in the *bla*_KPC_ gene. KP357 strain carried an intact *bla*_KPC-2_ gene, while KP697 strain had a mutated *bla*_KPC_ gene, named *bla*_KPC-71_. Compared to *bla*_KPC-2_, the *bla*_KPC-71_ gene showed a mutated nucleotide (A to C) and an insertion of 3 nucleotides at positions 542 to 545, which resulted in a variant with the subsequent insertion of a serine between amino acid sequence positions 182 and 183 (Fig. 1).

**TABLE 1:** Antibiotic susceptibility of the strains used in this study^*a*^

Strain^*c*^	MIC (mg/liter) for^*b*^:										
	AMC	FEP	CAZ	ETP	IPM	MEM	AMK	CIP	TGC	CST	CZA
E. coli DH5α	8	0.06	0.25	0.008	0.25	0.03	2	<0.06	0.25	0.125	0.25
E. coli DH5α/pCR2.1	32	0.5	1	0.015	0.5	0.03	2	<0.06	0.125	<0.125	0.25
E. coli DH5α/pKPC-2	128	>128	64	64	64	16	4	<0.06	0.125	0.125	0.25
E. coli DH5α/pKPC-71	64	8	64	0.125	0.25	<0.125	2	<0.06	0.125	0.25	16
K. pneumoniae KP357	128	>128	>128	>128	128	>128	64	>64	2	0.25	4
E. coli DH5α/pKP357	64	>128	>128	>128	64	64	64	<0.06	0.125	0.125	0.5
K. pneumoniae KP697	64	>128	>128	8	4	2	128	>64	1	0.25	>128
E. coli DH5α/pKP697_3	16	128	>128	0.25	0.5	<0.125	128	<0.06	0.125	<0.125	8
E. coli ATCC 25922	8	0.06	0.125	0.008	0.125	0.015	0.5	0.125	0.06	0.25	0.25

a Avibactam was added at 4 mg/liter.

b AMC, amoxicillin-clavulanic acid; FEP, cefepime; CAZ, ceftazidime; ETP, ertapenem; IPM, imipenem; MEM, meropenem; AMK, amikacin; CIP, ciprofloxacin; TGC, tigecycline; CST, colistin; CZA, ceftazidime-avibactam.

c E. coli DH5α/pCR2.1, E. coli DH5α was transformed by expression plasmid pCR2.1-TOPO as a control. E. coli DH5α/pKPC-2, E. coli DH5α was transformed by pKPC-2 plasmid carrying wild-type *bla*_KPC-2_ gene from K. pneumoniae KP357. E. coli DH5α/pKPC-71, E. coli DH5α was transformed by pKPC-71 plasmid carrying *bla*_KPC-71_ gene. E. coli DH5α/pKP697_3, E. coli DH5α was transformed by wild-type plasmid carrying *bla*_KPC-71_ gene from K. pneumoniae KP697. E. coli DH5α/pKP357, E. coli DH5α was transformed by wild-type plasmid carrying *bla*_KPC-2_ gene from K. pneumoniae KP3577.

In addition, the OmpK35- and OmpK36-encoding genes were both found without known mutations associated with CZA resistance.

### Genetic context of *bla*_KPC-71_-carrying plasmid.

The *bla*_KPC-71_ gene was located on a plasmid (pKP697_3, ∼130 kb) by S1-pulsed-field gel electrophoresis followed by Southern blotting data not shown). DH5α/pKP697_3 harboring the *bla*_KPC-71_ gene showed a CZA MIC of 8 mg/liter (32-fold increase compared with that of E. coli DH5α), indicating that the *bla*_KPC-71_-carrying plasmid could transfer its CZA-resistant phenotype into E. coli strain DH5α (Table 1).

**FIG 2:**
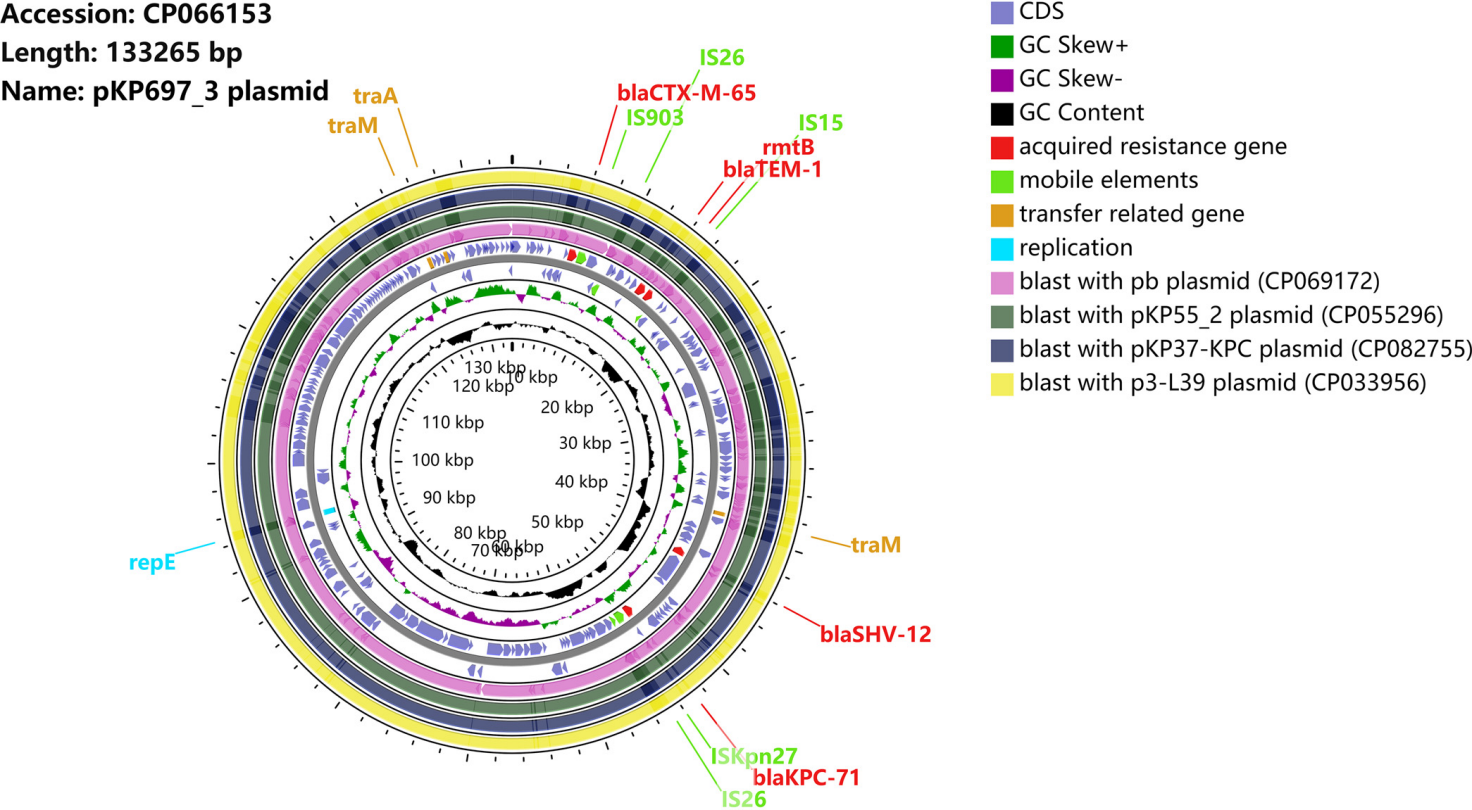
Schematic map of plasmid pKP697_3. Sequence alignment of plasmid pKP697_3 with sequences of the plasmids pKP55_2 (accession number CP055296), pb (accession number CP069172), pKP37-KPC (accession number CP082755), and p3-L39 (accession number CP033956). GC content is the inner circle in black.

The complete plasmid sequence was further acquired to characterize the plasmid. Sequence analysis revealed that the plasmid was 133,265 bp in length with 53.3% G+C content and harbored 208 predicted open reading frames (Fig. 2). The mutant *bla*_KPC-71_ gene was preceded by IS*26* and IS*Kpn27*, followed by IS*26*. In this plasmid, several antimicrobial resistance genes were also identified, such as *bla*_TEM-1_, *bla*_SHV-12_, *bla*_CTX-M-65_, and *rmtb*. Further sequence alignments revealed that the plasmid sequence showed almost identical nucleotide sequences (100% coverage and 99.99% identity) with the plasmids pKP55_2 (accession number CP055296) (15), pB (accession number CP069172), pKP37-KPC (accession number CP082755), and p3-L39 (accession number CP033956), indicating that *bla*_KPC-71_ gene mutation was derived from the *bla*_KPC-2_ gene based on the similarity of plasmid sequences.

### Identification of the KPC-71 enzyme involved in CZA resistance.

To confirm that the CZA resistance phenotype was mediated by the *bla*_KPC-71_ gene, cloning and expression experiments were performed. As we expected, the CZA MIC of E. coli DH5α/pKPC-2 expressing *bla*_KPC-2_ was 0.25 mg/liter. In contrast, the CZA MIC of E. coli DH5α/pKPC-71 expressing *bla*_KPC-71_ was 16 mg/liter (Table 1). In addition, the CZA MIC of wild-type E. coli DH5α and the vector-carrying strain E. coli DH5α/pCR2.1 was 0.25 mg/liter. Overall, these results demonstrated that the *bla*_KPC-71_ gene could result in a 64-fold increase in the MIC value. In addition, *bla*_KPC-71_ conferred susceptibility to carbapenems (Table 1).

### Enzyme kinetic data.

To understand the mechanism of CZA resistance, the enzyme kinetics of KPC-2 and KPC-71 were determined. The *k*_cat_/*K_m_* of the KPC-71 enzyme with ceftazidime was 4-fold lower than that of wild-type KPC-2. KPC-71 with ceftazidime exhibited a lower (∼13-fold) *K_m_* than wild-type KPC-2, indicating that compared with wild-type KPC-2, the KPC-71 enzyme showed decreased hydrolysis and higher affinity of ceftazidime (Table 2). In addition, the KPC-71 enzyme displayed a lower hydrolysis activity of nitrocefin (∼4,850-fold) than wild-type KPC-2 (Table 2). Notably, the KPC-71 enzyme has almost no detectable hydrolysis activity of carbapenems (ertapenem, imipenem, and meropenem) under our conditions (Table 2) (15), indicating that reduced hydrolysis activity caused by KPC-71 leads to susceptibility to carbapenems.

**TABLE 2:** Kinetic parameters of purified β-lactamases KPC-2 and KPC-71^*a*^

β-Lactam	KPC-2			KPC-71		
	*K_m_* (μM)	*k*_cat_ (s^−1^)	*k*_cat_/*K_m_* (μM^−1^·s^−1^)	*K_m_* (μM)	*k*_cat_ (s^−1^)	*k*_cat_/*K_m_* (μM^−1^·s^−1^)
Nitrocefin	22	139	6.3	32	0.043	0.0013
Ceftazidime	216	1.7	0.008	17	0.039	0.002
Meropenem	17	5.1	0.3	ND	ND	ND
Imipenem	220	59	0.27	ND	ND	ND
Ertapenem	15	6.9	0.46	ND	ND	ND

a ND, not determined due to a low initial rate of hydrolysis. *k*_cat_, turnover; *K_m_*, Michaelis constant (affinity); *k*_cat_/*K_m_*, specificity constant (hydrolysis).

To evaluate the inhibitory activity of the inhibitor against the KPC-2 and KPC-71 enzymes, the 50% inhibitory concentration (IC_50_) values of avibactam, tazobactam, and clavulanic acid were further measured (Table 3). Avibactam against KPC-71 exhibited an ∼14-fold higher IC_50_ value than wild-type KPC-2, indicating that KPC-71 was associated with low affinity and consequently reduced sensitivity to avibactam. In contrast, tazobactam and clavulanic acid against KPC-71 exhibited ∼3-fold and ∼2-fold lower IC_50_ values, respectively, than those against KPC-2.

**TABLE 3:** IC_50_ of β-lactamase inhibitors against KPC-2 and KPC-71^*a*^

Inhibitor	IC_50_ (μM)	
	KPC-2	KPC-71
Avibactam	0.034	0.47
Tazobactam	1.7	0.65
Clavulanic acid	0.66	0.42

a IC_50_ represents the concentration of a drug that is required for 50% inhibition of the enzymatic activity.

In short, these results indicate that CZA resistance caused by the 182S insertion observed in the KPC-71 sequence was involved in a higher affinity toward ceftazidime and in a reduced sensitivity to avibactam.

### Fitness effects of *bla*_KPC_ variants.

**FIG 3:**
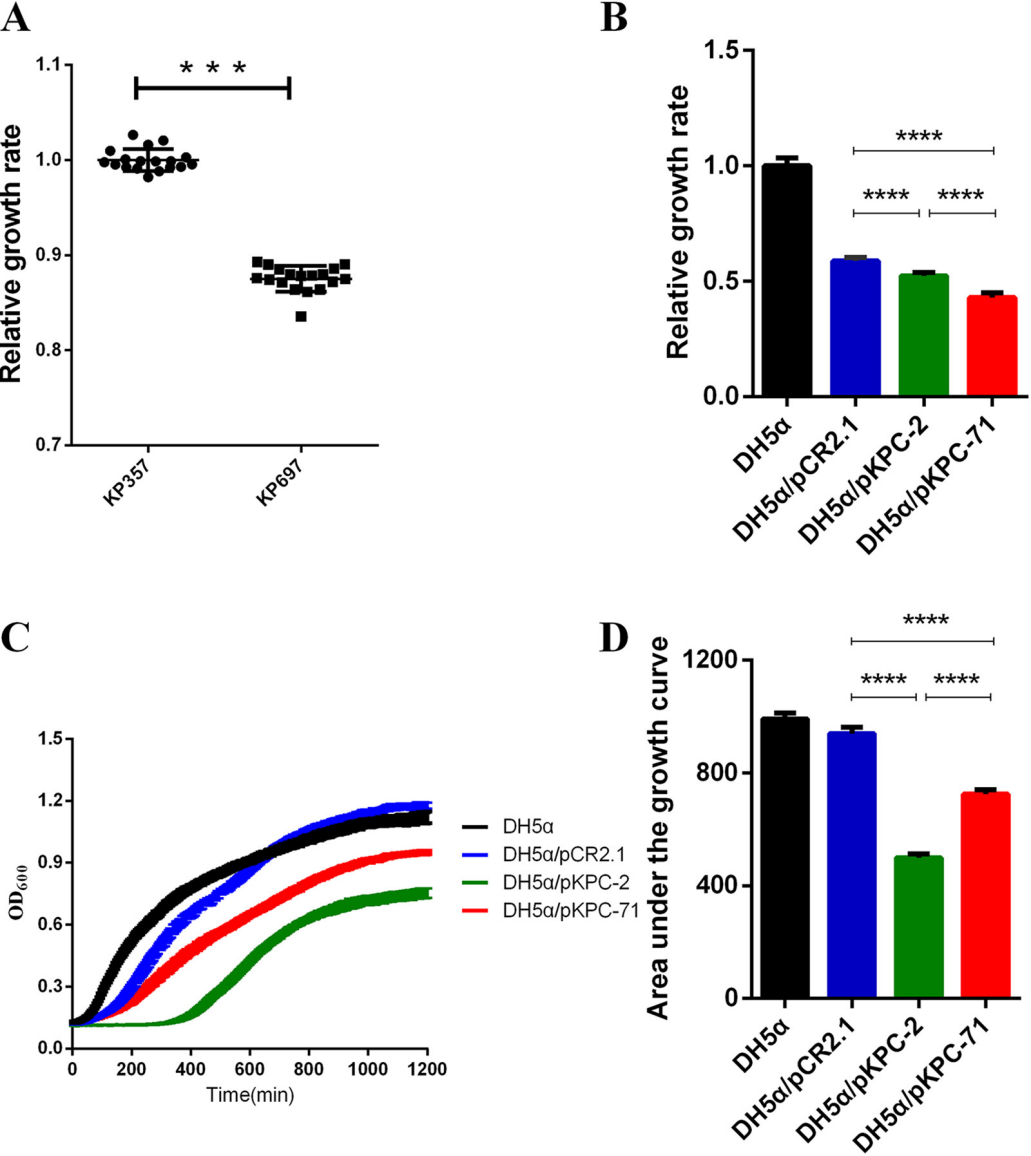
Growth conditions of isolated strains and constructed strains. (A) The growth rate of KP357 was normalized to 1, and the relative growth rates of CZA-susceptible isolate KP357 and CZA-resistant isolate KP697 are shown as means ± standard deviations. Each point represents a data value. (B to D) The relative growth rate (B), the growth curve (C), and the area under the growth curve (D) of constructed strains are presented. The growth rate of E. coli DH5α was normalized to 1. All experiments were performed with at least three independent replicates with three technical replicates in MH broth. Error bars represent the standard deviations. Statistical analysis was performed through unpaired *t* tests with two-way analysis of variance (ANOVA) and Tukey’s honestly significant difference (HSD) analysis for unequal variances. ***, *P* < 0.001; ****, *P* < 0.0001.

To investigate the effects of the *bla*_KPC_ variants on growth, we obtained the growth curves of the CZA-susceptible (KP357) and CZA-resistant (KP697) isolates in the absence of antibiotics. Results showed that the CZA-resistant isolate exhibited a significantly decreased growth rate compared to the CZA-susceptible isolate (Fig. 3A). To further evaluate the influence caused by the *bla*_KPC-71_ gene, we compared three bacterial growth parameters (the growth curve, the area under the growth curve [AUC], and the relative growth rate) of constructed strains in the absence of antibiotics to reflect fitness. The AUCs and the relative growth rates of DH5α/pKPC-2 strain and DH5α/pKPC-71 strain were significantly lower than those of the vector-carrying strain DH5α/pCR2.1, and the growth curves of both strains also indicated a growth disadvantage compared to DH5α/pCR2.1 (Fig. 3B to D). Thus, expression of both *bla*_KPC-2_ and *bla*_KPC-71_ genes has a negative impact on fitness.

Interestingly, we observed that the AUC value of the DH5α/pKPC-71 strain was significantly higher than that of the DH5α/pKPC-2 strain, but it exhibited a lower growth rate simultaneously. Overall, our results suggested that *bla*_KPC-71_ and *bla*_KPC-2_ affect different bacterial growth parameters, and the *bla*_KPC-71_ gene appeared to mainly retard the bacterial growth rate.

## DISCUSSION

KPC-2-producing ST11-type CRKP strains have been demonstrated to be a successful clonal lineage in China and to pose a threat in clinical settings (16–18). As one of the new therapeutic alternatives against KPC-2-producing CRKP strains, CZA has been widely used in China since its approval on 21 May 2019 (https://www.nmpa.gov.cn/). However, CZA resistance has been reported along with increasing usage of this drug in CRKP-infected patients (11). In this study, the two K. pneumoniae strains both belonged to the ST11 clonal lineage. Various resistance genes were detected in the two strains based on the whole-genome analysis. Moreover, the KP697 strain was resistant to CZA after exposure to CZA, indicating that ST11-type CRKP had a strong ability to acquire resistance. In addition, the CZA resistance gene was located in the plasmid, which may also lead to the further dissemination of CZA resistance to other bacteria. Therefore, effective measures must be immediately taken to prevent the spread of this resistance plasmid.

CZA resistance in CRKP strains is usually associated with a variety of mutations in the *bla*_KPC_ gene (13). The KPC enzyme has 4 loops surrounding the core of the active site (14). Amino acid substitutions in KPC-2 corresponding to the omega-loop of the protein, particularly at the Ambler 179 position, are mainly responsible for CZA resistance (11, 14). To date, 90 variants of the *bla*_KPC_ gene have been reported, more than 30 of which are CZA resistant. However, amino acid mutations located outside the loop region of KPC that result in resistance to CZA are rarely reported. In this study, we describe a novel KPC variant during CZA treatment of CRKP infections due to the change from KPC-2 to KPC-71 carbapenemase in China. The mechanism of resistance to CZA is due to a 182S insertion observed in the KPC-71 sequence. The kinetic parameters showed that KPC-71 exhibited higher affinity than wild-type KPC-2 toward ceftazidime and reduced sensitivity to avibactam. Increased ceftazidime affinity has been observed in KPC loop variants resulting in CZA resistance, such as the KPC-2 derivative (Asp179 Asn) (19) and KPC-3 derivatives KPC-3 (269-Pro-Asn-Lys-270 insertion) (20) and KPC-3 (276-Glu-Ala-Val-277 insertion) (21). In contrast to these previous studies, the KPC-71 mutation is located outside the loop region of KPC, indicating that CZA resistance mechanisms caused by KPC mutations are diverse. In addition, the reduced hydrolytic activity caused by KPC-71 restored the susceptibility to carbapenems.

Notably, resistance mutations have been observed in patients being treated with CZA. Resistance has been found to develop in KPC-2- or KPC-3-producing K. pneumoniae isolates during therapy (17–20). A recent molecular epidemiology study reported that up to 48.4% (15/31) of clinical CZA-resistant K. pneumoniae isolates caused by various mutations were isolated after treatment with CZA (22). Our study further confirmed that CZA resistance caused by a mutated *bla*_KPC_ gene was selected after CZA therapy.

Antibiotic resistance emergence is associated with the influence of strain growth (23). In this study, both *bla*_KPC-2_ and *bla*_KPC-71_ gene expression have a negative impact on fitness. Interestingly, the AUC value of the DH5α/pKPC-71 strain was significantly higher than that of the DH5α/pKPC-2 strain. CZA is currently an important option for the treatment of KPC-producing K. pneumoniae bacteria. However, during treatment, bacterial resistance to CZA caused by mutations of the *bla*_KPC_ gene should receive clinical attention. The risk of the development of CZA resistance in CRKP strains under treatment pressure should be a major concern.

CARBA 5, a commercial rapid detection method for KPC-producing strains, displayed a failure of detection for the KP697 strain producing KPC-71 (data not shown). 182S insertion of KPC-2 probably resulted in KPC-2 protein structure change (see Fig. S1 in the supplemental material) and might fail to combine the KPC-2 protein antibody. Similar results were also observed in our previous study (15). However, the precise role of this 182S insertion outside the omega loop remains unclear and will need further studies. Notably, currently there is no molecular test to detect CZA resistance. Therefore, improving molecular screening is imperative to detect *bla*_KPC_ mutations rapidly and accurately.

10.1128/mSphere.00859-21.1 FIG S1 Crystal structure of KPC-2. The crystal structure of KPC-2 (PDB entry 5UJ3) was analyzed by the PyMOL Molecular Graphics System. KPC-2 is shown as a backbone ribbon. The omega-loop is colored in red. For KPC-71, one amino acid is inserted at the position of the black arrow. Download FIG S1, PDF file, 0.3 MB. Copyright © 2021 Li et al. 2021 Li et al. https://creativecommons.org/licenses/by/4.0/ This content is distributed under the terms of the Creative Commons Attribution 4.0 International license.

### Conclusions.

Our findings are notable for several reasons. First, we reported an ST11-type clinical CRKP isolate that produces KPC-71, a novel plasmid-borne KPC variant that confers CZA resistance. Second, we proved that resistance to CZA is mediated by the 182S insertion in the KPC enzyme, which increases ceftazidime affinity and decreases avibactam inhibition. Third, we found that expression of both *bla*_KPC-2_ and *bla*_KPC-71_ genes has a negative impact on fitness, *bla*_KPC-71_ and *bla*_KPC-2_ might affect different bacterial growth parameters, and the *bla*_KPC-71_ gene appeared to mainly retard the bacterial growth rate.

Considering the wide application of CZA, clinicians should pay attention to the risk of the development of CZA resistance in CRKP strains under treatment pressure.

## MATERIALS AND METHODS

### Patient and isolate data.

The patient was a 22-year-old male. He had suffered from brain damage due to accidental falling on April 2020. During hospitalization, the patient developed ventilator-associated pneumonia, and a CRKP (KP357, *bla*_KPC-2_ positive) was isolated from sputum. KP357 was susceptible to CZA (4 mg/liter). After receiving 2.5 g CZA three times a day for 2 weeks, the patient's symptoms were improved. Ten days later, this patient had a fever accompanied by increased CRP (C-reactive protein). The second K. pneumoniae strain, named KP697, was isolated from blood culture, and this strain was resistant to CZA (>128 mg/liter). The two strains were both preliminarily identified by the matrix-assisted laser desorption ionization–time of flight mass spectrometry system (bioMérieux, Marcy l'Etoile, France) and further confirmed by whole-genome sequencing.

E. coli DH5α and E. coli BL21 were used in the genetic procedures.

### Antimicrobial susceptibility testing.

MICs were determined according to the reference Clinical and Laboratory Standards Institute (CLSI) broth microdilution method (24). The MICs were interpreted according to CLSI guidelines, except those for tigecycline and colistin, which were interpreted according to the European Committee on Antimicrobial Susceptibility Testing (EUCAST) criteria for *Enterobacteriaceae* (http://www.eucast.org/clinical_breakpoints). E. coli ATCC 25922 was used as a quality control strain.

The type of carbapenemase enzyme was investigated by NG-Test Carba 5 (Biotech).

### Plasmid transformation experiments.

Plasmid extraction and transformation were performed according to our previous study (25). Briefly, plasmid DNA was obtained by a Qiagen plasmid midi kit (Qiagen, Germany) and then was electrotransformed into E. coli DH5α. Mueller-Hinton (MH) agar plates containing ampicillin (100 mg/liter) was used to select the transformants, which were further confirmed by PCR sequencing and antimicrobial susceptibility testing.

### Genomic DNA extraction, analysis, and comparison.

The genomic DNA of the K. pneumoniae KP357 and KP697 strains was extracted using a QIAamp DNA minikit (Qiagen, Valencia, CA, USA) by following the manufacturer’s recommendations. The genomes were sequenced on an Illumina-HiSeq X-10 (Illumina Inc., San Diego, CA) and the MinION platform (Nanopore, Oxford, UK) to acquire the complete chromosomes and plasmid sequences, respectively. Sequence reads were assembled using the CLC Genomics Workbench software package (CLC Bio 10.0) and Unicycler version 0.4.8.25. Annotation of the plasmid genomes was performed using the Rapid Annotation using Subsystems Technology (RAST) annotation website server (https://rast.nmpdr.org/). Comparison between the pKP697_3 plasmid sequence (accession number CP066153) and the reference plasmid pKP55_2 (accession number CP055296) was performed by GC viewer (26).

To explore the mechanism of CZA resistance, whole-genome sequences were compared between CZA-susceptible and CZA-resistant isolates using Snippy V4.4.5 (https://github.com/tseemann/snippy) and breseq v0.33.0 (27).

### Cloning experiments.

Cloning experiments were performed as previously described (28). Briefly, the wild-type *bla*_KPC-2_ gene and *bla*_KPC-71_ gene sequences containing the wild promoter were amplified from the K. pneumoniae KP357 and KP697 strains, respectively. The PCR products were purified and then cloned into the pCR2.1-TOPO vector (Invitrogen, Shanghai, China). The recombinant plasmids, pKPC-2 and pKPC-71, were both introduced into the E. coli DH5α strain via chemical transformation experiments. MH agar plates including 50 mg/liter kanamycin were used to select transformants, which were further verified by PCR and sequencing.

### *In silico* analysis.

Acquired resistance genes, Inc-type plasmids, and multilocus sequence typing (MLST) of the strains were obtained by the ResFinder 4.1, Plasmid Finder 1.3, and MLST 2.1 servers, which are available at the Center for Genomic Epidemiology (http://www.genomicepidemiology.org/). The crystal structure of KPC-2 (PDB entry 5UJ3) was retrieved from the Protein Data Bank and further analyzed by the PyMOL Molecular Graphs System (http://pymolwiki.org/PLoS).

### Steady-state enzyme kinetic measurements.

Cloning, expression, and purification of β-lactamase were performed as previously described (15). Protein concentration was determined by absorbance at 280 nm using an extinction coefficient of 39,545 M^−1^ cm^−1^. Kinetic parameters of purified enzymes were measured by spectrophotometry at room temperature and in PBS at pH 7.4, and then *K_m_* and *k*_cat_ were obtained by Michaelis-Menten equation fitting (19). For nitrocefin, the values of *K_m_* and *k*_cat_ were obtained by measuring the initial velocities of various concentrations of nitrocefin, and the substrate cleavage was monitored at 482 nm.

To evaluate the kinetic parameters of ceftazidime, the enzyme was mixed with substrates of various concentrations, and the cleavage level of the substrates was monitored at 257 nm at room temperature. The initial cleavage rate of ceftazidime was calculated. Similarly, the kinetic parameters of ertapenem, imipenem, and meropenem were calculated using the same method, but the substrate cleavage was determined at 297 nm, 299 nm, and 299 nm, respectively.

IC_50_ values of inhibition of wild-type KPC-2 and KPC-71 protein by avibactam, tazobactam, and clavulanic acid with nitrocefin as the substrate were determined. Enzymes were mixed with these inhibitors at concentrations varying from 0 to 100 μM in PBS and incubated for 10 min, and ceftazidime was subsequently added at a concentration of 100 μM. Absorbance was recorded at 482 nm after 30 min and exported to Prism software to calculate IC_50_ values using the dose-response-inhibition, variable-slope (four parameters) equation.

### Fitness evaluation.

The growth curve, the area under the curve (AUC), and the relative growth rate were used as indicators of fitness and were assessed as previously described (29). Four independent cultures of each strain were grown overnight in MH broth. These cultures were diluted 1:100, and 200 μl of diluted culture was added into a flat-bottom 100-well plate in four replicates at 37°C with shaking. The optical density at 600 nm (OD_600_) of each culture was determined every 5 min for 20 h using a Bioscreen C Analyzer (Oy Growth Curves Ab. Ltd., Finland). The growth rate based on OD_600_ curves was calculated by an R script (30). Growth curves were constructed and used to calculate AUC by GraphPad Prism 8. Two-way analysis of variance (ANOVA) and Tukey’s honestly significant difference (HSD) analyses were used to evaluate differences between the means, with a significant probability at a *P* value of ≤0.05.

### Ethics approval.

This study was conducted in accordance with the Declaration of Helsinki and was reviewed and approved by the Research Ethics Committee of the First Affiliated Hospital of Zhejiang University (IIT20210268A).

### Data availability.

The complete genome sequences of K. pneumoniae KP697 and KP357 reported in the present study were deposited in the GenBank nucleotide database under accession numbers CP066151–CP066155 and JAHSUF000000000. In addition, the *bla*_KPC-71_ gene was deposited in the NCBI database under accession no. MW015092.
