# Supplementary figures and images for: Molecular Mechanisms Driving the In Vivo Development of KPC-71-Mediated Resistance to Ceftazidime-Avibactam during Treatment of Carbapenem-Resistant Klebsiella pneumoniae Infections
Source: mSphere. 2021 Dec 22;6(6):e00859-21. doi: 10.1128/mSphere.00859-21 (PMC8694138; doi:10.1128/mSphere.00859-21)

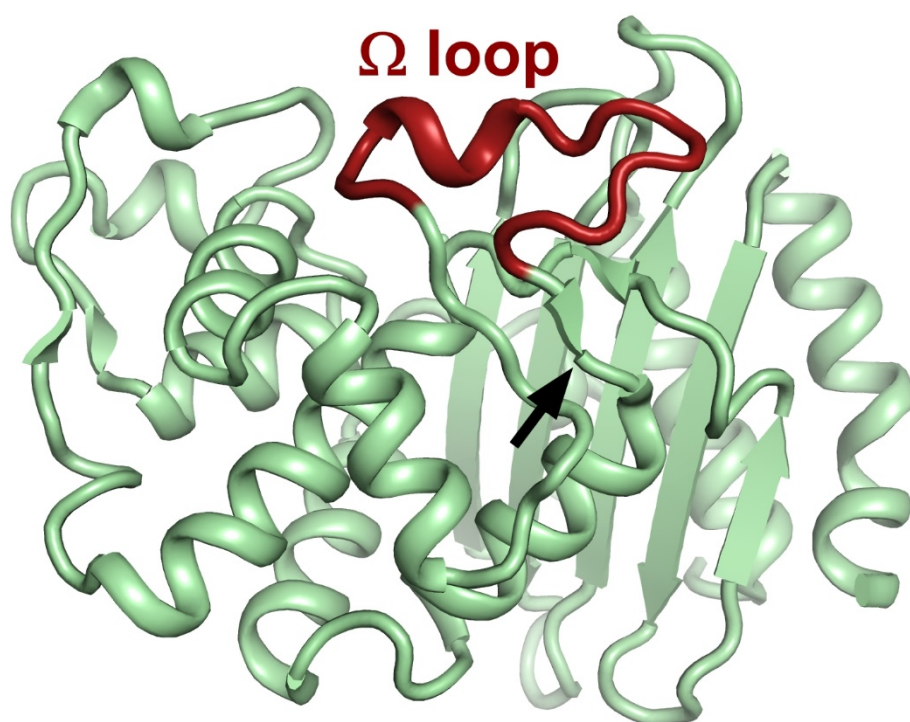

Supplement: FIG S1 [file msphere.00859-21-sf001.pdf]
